# *Datura stramonium* Leaf Extract Exhibits Anti-inflammatory Activity in CCL_4_-Induced Hepatic Injury Model by Modulating Oxidative Stress Markers and iNOS/Nrf2 Expression

**DOI:** 10.1155/2022/1382878

**Published:** 2022-03-16

**Authors:** Bakht Nasir, Ashraf Ullah Khan, Muhammad Waleed Baig, Yusuf S. Althobaiti, Muhammad Faheem, Ihsan-Ul Haq

**Affiliations:** ^1^Department of Pharmacy, Faculty of Biological Sciences, Quaid-i-Azam University, Islamabad 45320, Pakistan; ^2^Faculty of Pharmaceutical Sciences, Abasyn University Peshawar, Peshawar 25000, Pakistan; ^3^Department of Pharmacology and Toxicology, College of Pharmacy, Taif University, P.O. Box 11099, Taif 21944, Saudi Arabia; ^4^Addiction and Neuroscience Research Unit, Taif University, P.O. Box 11099, Taif 21944, Saudi Arabia; ^5^Riphah Institute of Pharmaceutical Sciences, Riphah International University, Islamabad 45320, Pakistan

## Abstract

**Background:**

Inflammation is a frequent phenomenon in the pathogenesis of hepatic disorders leading to fibrosis and cirrhosis. Phytopharmaceuticals developed from traditional medicine can provide effective therapeutic alternatives to conventional medications. *Datura stramonium* (DS) has reported traditional uses in inflammatory diseases. In this study, we have tried to validate its potential as a source of anti-inflammatory agents.

**Methods:**

Powdered leaf part of DS was extracted using ethyl acetate (EA) to provide the extract (DSL-EA). Lymphocyte and macrophage viability and acute toxicity assays established the safety profile, while nitric oxide (NO) scavenging assay estimated the *in vitro* anti-inflammatory potential. Noninvasive anti-inflammatory, antidepressant, and antinociceptive activities were monitored using BALB/c mice using low and high doses (150 and 250 mg/kg). Major inflammatory studies were performed on Sprague-Dawley male rats using CCl_4_-induced liver injury model. Disease induction was initiated by intraperitoneal injections of CCl_4_ (1 mL/kg of 30% CCl_4_ in olive oil). The rats were divided into six groups. The anti-inflammatory potential of DSL-EA in low and high doses (150 and 300 mg/kg, respectively) was assessed through hematological, biochemical, liver antioxidant defense, oxidative stress markers, and histological studies as well as the expression of Nrf2 and iNOS.

**Results:**

DSL-EA exhibited prominent *in vitro* NO scavenging (IC_50_: 7.625 ± 0.51 *μ*g/mL) and *in vivo* anti-inflammatory activity in paw and anal edema models. In CCl_4_ model, hematological investigations revealed vasotonic effects. Liver functionality was significantly (*P* < 0.001 − 0.05) improved in DSL-EA-treated rats. The activity level of endogenous antioxidant enzymes in liver tissues was improved in a manner identical to silymarin. The extract reduced the percent concentration of oxidative stress markers in liver tissues. Furthermore, DSL-EA displayed restorative effects on histological parameters (H and E and Masson's trichrome staining). Immunohistochemistry studies showed marked decline in Nrf2 expression, while overexpression of iNOS was also observed in disease control rats. The damage was distinctly reversed by the extract.

## 1. Introduction

Oxygen and nitrogen are found abundantly in any aerobic system. These molecules are involved in numerous physiological and metabolic processes, and they undergo chemical changes within the system [1]. These molecules go into a readily reactive state when these are transformed into unpaired moieties within the body. Few examples of such reactive species are singlet oxygen, superoxide anion, nitric oxide, hydroxyl radical, etc. [2]. The terms reactive oxygen species (ROS) and reactive nitrogen species (RNS) were used to describe these moieties since there are numerous free oxygen and nitrogen radicals and nonradicals involved in the overall process. These molecules are formed within the living system as a result of various exogenous and endogenous factors [3]. Generation of these species in appropriate amount plays a key role in normal physiological functions, i.e., body's defense against microbes and signal transduction, but the problem arises when these are produced in excessive amounts ultimately resulting in oxidative stress. These molecules, due to their chemical nature, are involved in DNA damage, lipid peroxidation, and oxidation of numerous cellular molecules including cellular membranes, the inevitable consequence being cellular injury [4]. Out of the many endogenous sources leading to generation of free radicals, drugs, pollutants, radiations, and chemicals are the most common ones [5]. The pathological conditions resulting from such oxidative insults include liver cirrhosis, cancer, Alzheimer's disease, diabetes mellitus, rheumatoid arthritis, and multiple sclerosis [6, 7]. The liver is not just responsible for metabolism but also the largest organ involved in detoxification and life sustainability [8]. Our body relies on the liver for breakdown of toxins produced inside the body or ingested from outside; this makes the liver particularly vulnerable to long-term exposure of oxidative stress [9]. Liver fibrosis is a serious health condition, and if not treated promptly, it leads to several liver ailments including liver cirrhosis and hepatocellular carcinoma (HCC) [10]. Different factors are responsible for the development and progression of liver diseases; the involvement of exogenous toxins and drugs cannot be overruled. Carbon tetrachloride (CCL_4_) has been widely employed as a toxin in liver injury models. The mode of action of CCL_4_-induced toxicity is production of oxidative stress leading to steatosis and centrilobular necrosis, while prolonged exposure causes chronic liver injury resulting in liver fibrosis [11].

Phytochemicals from different medicinal plants are known to possess significant antioxidant properties. Some of the important antioxidant phytochemicals include phenolics and flavonoids, terpenoids, vitamins, alkaloids, saponins, minerals, and certain pigments. These diverse groups of compounds help the body to combat various oxidative stress-mediated diseases [12, 13]. Antioxidants protect the body tissues by scavenging the highly reactive oxygen compounds [14]. Apart from the antioxidant nature of plants, the underlying mechanisms of the protective action against liver injuries may include their anti-inflammatory, antinecrosis, and regulatory action on lipid metabolism as well as their antiapoptotic effects [15]. Genus *Datura* (family Solanaceae) consists of medicinally important species including but not limited to *D. stramonium*, *D. ferox*, *D. innoxia*, and *D. metel* [16]. These species are being used for medicinal and recreational purposes since time immemorial. *Datura* species have several traditional and ethnopharmacological uses, i.e., antiasthmatic, anesthetic, sedative, antihemorroidal, expectorant, demulcent, and antitumor [16, 17]. *D. stramonium* Linn. is the most common species within genus *Datura* native to Asia but found abundantly in tropical, subtropical, and temperate regions around the world [18]. It is well recognized as a valuable remedy in numerous ailments including inflammation, rheumatoid arthritis, wounds, ulcers, gout, asthma and bronchitis, fever, and toothache [19]. *D. stramonium* is chosen for the current study because of ample evidence in the literature substantiating its potential as a source of anti-inflammatory agents [20]. Our study aims to look for a scientific validation of the ethnomedicinal use of *D. stramonium* extracts in inflammatory disorders using acute and chronic *in vivo* models. This study will provide a comprehensive insight into the potential of selected plant extract in alleviating liver inflammation and its role in modulating the oxidative stress markers.

## 2. Results

In the previously published study covering the initial phase of our project, the ethyl acetate leaf extract of *D. stramonium* (DSL-EA) was qualitatively and quantitatively evaluated for the presence of pharmacologically significant secondary metabolites including flavonoids and phenolics. Several *in vitro* antioxidant and anticancer studies were also undertaken which substantiated the medicinal worth of the selected plant species [16]. In this study, the anti-inflammatory potential of DSL-EA is appraised. The details of a series of assays performed to scientifically validate the folkloric use of this medicinal plant are given in the following sections.

### 2.1. Nitric Oxide Scavenging Potential

Nitric oxide scavenging action was determined to further establish the efficacy profile of selected extract before initiating the acute and sub chronic *in vivo* studies. The highest concentration of DLS-EA used in this assay was 20 *μ*g/mL, and it inhibited NO production to a significant level. The response tapered down at lower concentrations proving a concentration-dependent scavenging response. Maximum % NO production of 67.62 ± 0.45% was observed at the lowest tested concentration of DSL-EA, i.e., 2.5 *μ*g/mL, while only 27.35 ± 0.12% NO production was estimated at 20 *μ*g/mL. The IC_50_ value recorded was 7.625 ± 0.51 *μ*g/mL.

### 2.2. Toxicity Studies

#### 2.2.1. Lymphocyte and Macrophage Toxicity Assay

Cytotoxicity against normal human lymphocytes was tested as reported in our previous study [16]. Cytotoxicity against isolated macrophages from rat peritoneum was also determined. The percent cell viability was determined by performing methyl thiazole tetrazolium (MTT) assay. The results were expressed in terms of % viable cells in the test wells having predetermined concentration of DSL-EA and designated controls. The results have shown that DSL-EA, even at a maximum concentration of 20 *μ*g/mL, resulted in cell viability greater than 90%.

### 2.3. Acute *In Vivo* Studies

#### 2.3.1. Acute Toxicity Study

Acute toxicity was checked after a single booster dose (150-2000 mg/kg) to rats divided into different groups. The rats were kept under surveillance for a period of two weeks. No deaths were recorded during the said time period, and the rats did not experience any abnormal changes in the behavior. Normal physiology and all the senses were intact which showed that DSL-EA was safe to be administered up to a highest dose of 2000 mg/kg with no risk of any kind of toxicity.

#### 2.3.2. Noninvasive *In Vivo* Tests

**Figure 1:**
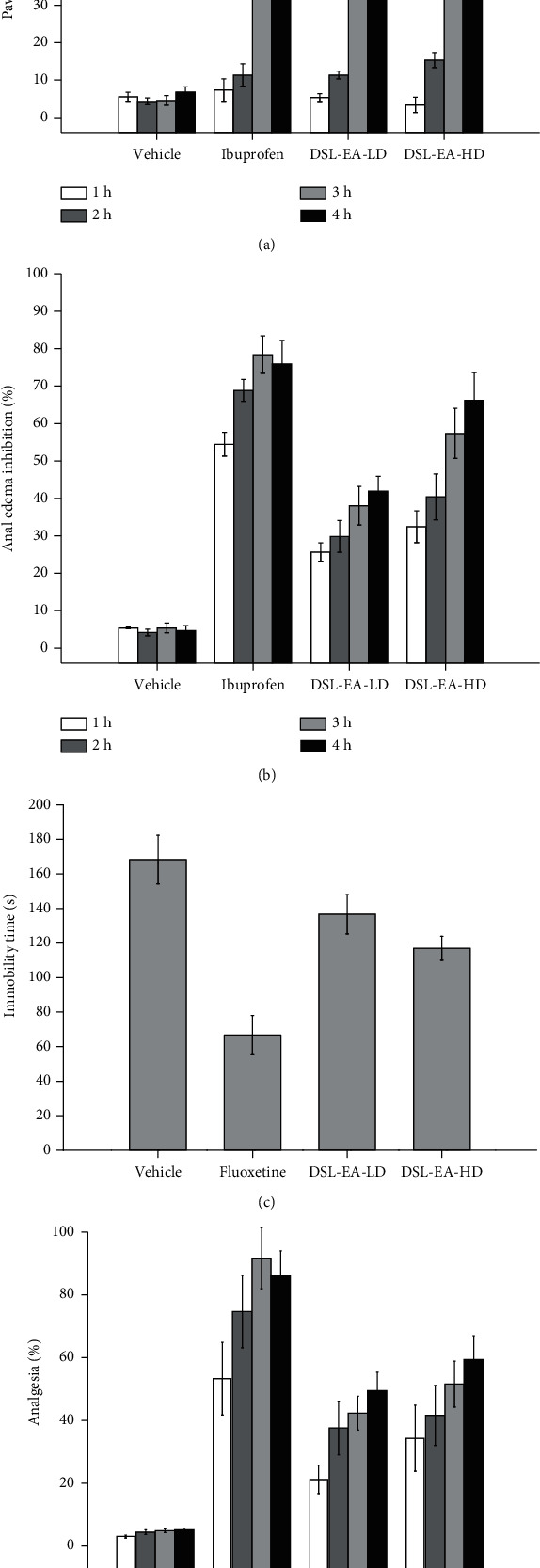
Percent paw edema inhibition (a), percent anal edema inhibition (b), immobility time in seconds (c), and percent analgesia (d) by DSL-EA extract in BALB/c mice. Data shown is presented as mean ± SD (*n* = 6). DSL-EA-LD: low-dose ethyl acetate extract of *D. stramonium*; DSL-EA-HD: high-dose ethyl acetate extract of *D. stramonium*.

*(1) Paw Edema*. The anti-inflammatory potential of DSL-EA was evaluated by calculating its edema inhibitory effect in carrageenan-induced hind paw edema test. As evident from the results in Figure 1, DSL-EA has alleviated the edema caused by carrageenan in a dose- and time-dependent manner with high dose of DSL-EA (300 mg/kg) showing more than 70% inhibitory effect. Low dose of the extract (150 mg/kg) exhibited moderate activity with the effect peaking at the 4^th^ hour, i.e., 51.63 ± 5.49% reduction noted in hind paw edema.

*(2) Anal Edema*. Croton oil-induced anal edema test was also performed for further validation of the anti-inflammatory potential of DSL-EA. A substantial steady decline in percent edema volume was observed over the course of the study duration, and maximum activity in terms of edema inhibition was observed at the 4^th^ hour in case of DSL-EA-HD, i.e., 67.62 ± 7.56%. Low dose of the extract curbed the edema by slightly less than 43% as of the 4^th^ hour reading. Ibuprofen was the standard drug used in the study, and it showed 80.1 ± 5.1% edema inhibition at the 3^rd^ hour. The details can be seen in Figure 1.

*(3) Tail Suspension Test*. The potential antidepressant action of extract was estimated using tail suspension test. Fluoxetine was the standard drug used in this assay, and it significantly reduced the immobility time in mice (66.66 ± 11.37 sec) in comparison to the vehicle control group (168.33 ± 14.04 sec). DSL-EA-HD reduced the immobility time only slightly (117 ± 6.93), and somewhat insignificant effect was observed in the case of DSL-EA-LD (136.66 ± 11.37). The results are stacked in Figure 1.

*(4) Hotplate Test*. The analgesic property of DSL-EA was also determined against thermally induced pain using hotplate method. The antinociceptive effect was evaluated by observing the % increase in latency period, and the results were compared with standard drug, i.e., tramadol. The maximum antinociceptive action of tramadol was observed at the 3^rd^ hour, i.e., 91.66 ± 9.74% increase in latency period. DSL-EA-HD moderately elevated the latency period with maximum increment of 59.4 ± 7.6% observed at the 4^th^ hour. DSL-EA-LD yielded slightly less significant response with a maximum of 49.5 ± 5.8% noted at the 4^th^ hour as shown in Figure 1.

### 2.4. Chronic *In Vivo* Study (CCL_4_-Induced Hepatic Injury in Sprague-Dawley Rats)

Following intraperitoneal administration of predetermined doses of CCL_4_ to the rats in groups III-VI (disease control, silymarin treated, DSL-EA low dose (LD), and DSL-EA high dose (HD)) and induction of hepatic toxicity, groups were treated with respective drugs and samples. The efficacy of low and high doses of DSL-EA alleviation of the hepatic toxicity was measured through extensive hematological, biochemical, and histological examinations. The effects produced by crude extract were compared with the controls used in the study.

#### 2.4.1. CCL_4_-Induced Hematological Variations

The results of CCL_4_-induced hematological variations are shown in Table 1. As obvious from the data, CCL_4_ has caused significant aberrations, i.e., decline in red blood cells (RBC) (6.07 ± 0.32 × 106/mm^3^) and hemoglobin (HGB) (8.02 ± 1.02 g/dL) while significant hike in white blood cells (WBC) (13.12 ± 0.03 × 103/mm^3^), neutrophils (62.04 ± 1.31%), monocytes (12.12 ± 1.03%), eosinophils (0.90 ± 0.22%), and basophils (0.81 ± 0.05%) in disease control rats. The hematological parameters of DSL-EA-treated groups are significantly different from the disease control group (*P* < 0.05, 0.01, and 0.001). DSL-EA crude extract normalized the aberrations in a dose-dependent manner fairly identical to silymarin given at 50 mg/kg dose.

#### 2.4.2. Effect on Biochemical Parameters

The deleterious effects of CCL_4_ on the liver and kidneys were further confirmed by numerous biochemical tests performed using serum acquired from rats of each study group. Liver function tests showed significantly higher level of liver enzymes (*P* < 0.05, 0.01, and 0.001) in comparison to other study groups. Alanine transaminase (ALT), aspartate aminotransferase (AST), alkaline phosphatase (ALP), and bilirubin levels recorded in serum of disease control rats were 188.73 ± 3.24, 231.98 ± 2.32, 172.74 ± 4.41 (U/L), and 2.99 ± 0.04 mg/dL, respectively. Furthermore, albumin level was markedly lower in the disease control group, i.e., 4.28 ± 0.09 g/L. Low and high doses of DSL-EA crude extract dose dependently reverted the harm done to liver functionality by CCL_4_ administration. High-dose treatment yielded significant alleviation (*P* < 0.05, 0.01, and 0.001) of the liver toxicity in a manner identical to positive control. Creatinine level was also elevated in the disease control group (1.08 ± 0.04 mg/dL). There was no statistically significant difference (*P* < 0.05, 0.01, and 0.001) between the positive control, DSL-EA-LD, and DSL-EA-HD groups when their serum creatinine levels were compared. The results are further elaborated in Table 2.

#### 2.4.3. Effect on Endogenous Antioxidant Enzymes and GSH Levels

**Figure 2:**
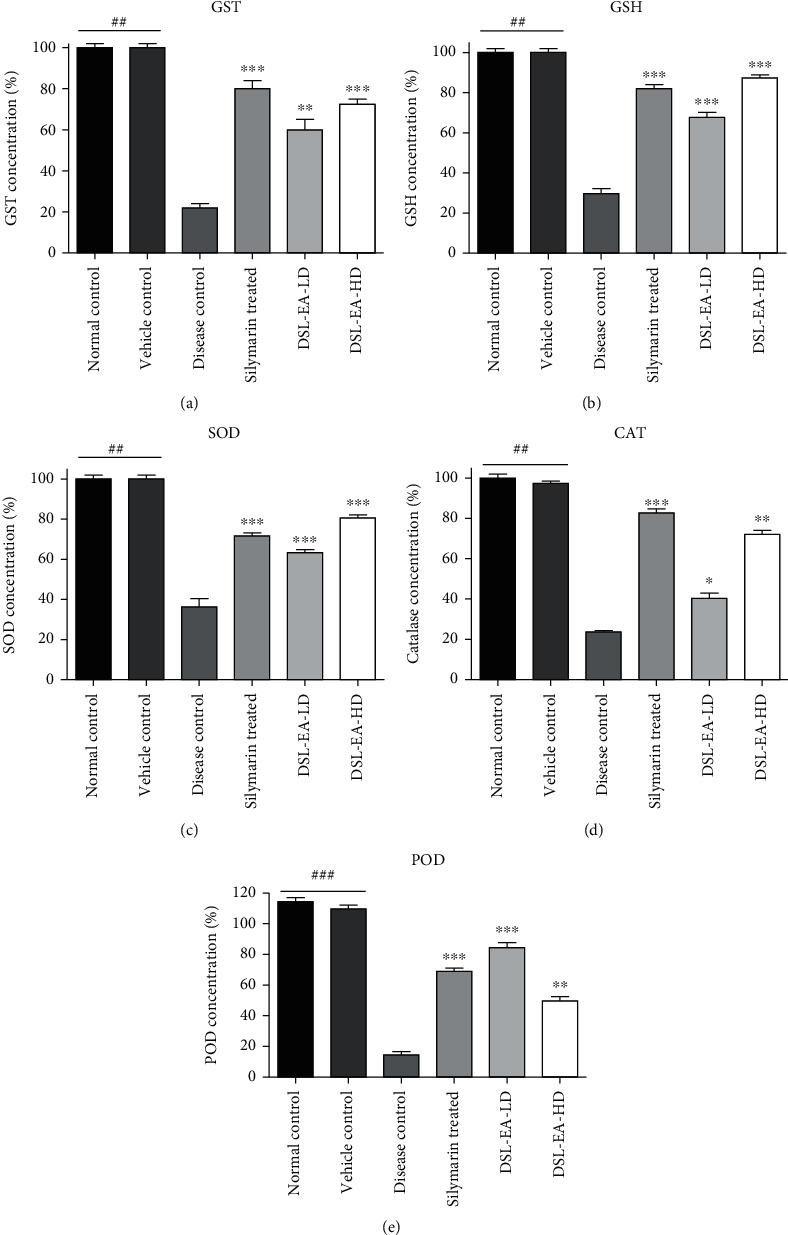
Effect of DSL-EA-LD and HD on glutathione S-transferase (GST) (a), glutathione (GSH) (b), superoxide dismutase (SOD) (c), catalase (d), and peroxidase (POD) (e) levels in liver tissue compared to the disease control group. All values are expressed as mean ± SEM (*n* = 6), ^∗^*P* < 0.05, ^∗∗^*P* < 0.01, and ^∗∗∗^*P* < 0.001 as compared to the disease control group.

The effect of DSL-EA treatment on endogenous antioxidant enzymes and glutathione (GSH) levels of the study groups is presented in Figure 2. A significant (*P* < 0.05, 0.01, and 0.001) reduction in activity level of glutathione S-transferase (GST), GSH, superoxide dismutase (SOD), catalase, and peroxidase (POD) was observed in the disease control group further confirming the damage inflicted by CCL_4_ dosing. High-dose treatments with DSL-EA curbed the damage and elevated the level of antioxidant enzymes and GSH to a similar extent as observed in silymarin-treated group. The % enzyme activity levels in liver tissues are presented in Figure 2.

#### 2.4.4. Effect on Oxidative Stress Markers

**Figure 3:**
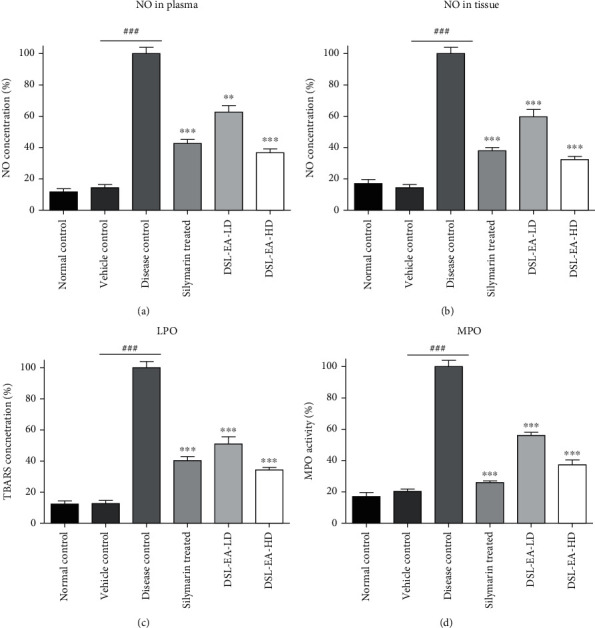
Effect of the DSL-EA-LD and HD on the oxidative stress markers such as nitric oxide (NO) in plasma (a), NO in liver tissue (b), malondialdehyde (MDA) (c), and myeloperoxidase (MPO) (d) concentration in liver tissues compared to the disease control group. All values are expressed as mean ± SEM (*n* = 6), ^∗^*P* < 0.05, ^∗∗^*P* < 0.01, and ^∗∗∗^*P* < 0.001. LPO: lipid peroxidation assay.

The oxidative stress markers investigated in the current study were NO, malondialdehyde (MDA), and myeloperoxidase (MPO), and their levels were greatly elevated in liver tissues of the disease control group. NO levels were assessed both in plasma and liver tissue since it is an important mediator of inflammation influenced by iNOS gene. As presented in Figure 3, % NO levels both in plasma and tissue homogenates were excessively elevated in the disease control group. DSL-EA-HD and silymarin decreased the NO levels in a significant (*P* < 0.05, 0.01, and 0.001) and comparable manner, while DSL-EA-LD treatments also yielded very good results.

There was statistically significant (*P* < 0.05, 0.01, and 0.001) difference between the % MDA level of the disease control rats and DSL-EA-treated groups. Dose-dependent decline in the oxidative stress was observed in treatment groups which further support the results of % enzyme activity and antioxidant status as discussed earlier. Low-dose treatment with DSL-EA also resulted in moderate improvement of the overall oxidative status of test rats. The details are presented in Figure 3.

The MPO expression levels in liver tissue act as marker of neutrophilic infiltration during inflammation. As expected in CCL_4_-induced liver injury, the MPO expression was markedly increased in the liver tissues of the disease control rats. Silymarin, DSL-EA-LD, and DSLA-EA-HD decreased the % MPO activity in a statistically significant and comparable manner (*P* < 0.05, 0.01, and 0.001) as shown in Figure 3. The reduced MPO activity greatly helps in curbing neutrophilic infiltration and inflammation in liver tissues.

#### 2.4.5. Effects on Histopathology (H&E and Masson's Trichrome Staining)

**Figure 4:**
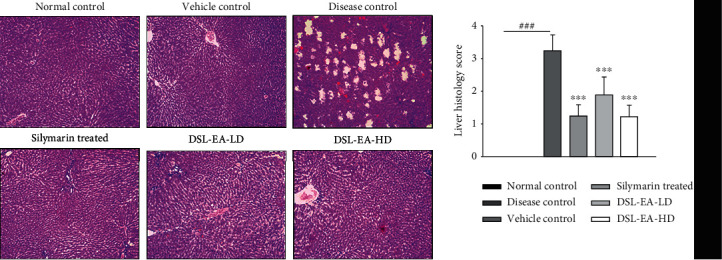
Hematoxylin-eosin (H and E) staining of the liver tissue. The restorative effect of the DSL-EA-LD and HD on liver tissues following CCL_4_-induced liver injury. The extracts markedly improved the histological parameters such as immune cell infiltration, fibrosis, and edema compared to the disease control. All values are expressed as mean ± SEM (*n* = 6), ^∗^*P* < 0.05, ^∗∗^*P* < 0.01, and ^∗∗∗^*P* < 0.001 as compared to the disease control group.

Histological examination has further ratified the findings of biochemical tests performed previously. Liver tissues of the normal and vehicle control groups were having normal morphological features, i.e., unharmed hepatocytes, central veins, and sinusoids. The disease control group incurred severe liver injury due to CCL_4_ injections. The most obvious signs of liver damage were immune cell infiltration, fibrosis, necrosed hepatocytes, and edema as evident in Figure 4 (hematoxylin-eosin (H&E) staining). In an identical manner to effects of DSL-EA observed in biochemical parameters, there was a significant (*P* < 0.05, 0.01, and 0.001) dose-dependent restorative effect on liver tissues. There was no significant difference between the liver histology score of silymarin and DSL-EA-HD-treated groups.

**Figure 5:**
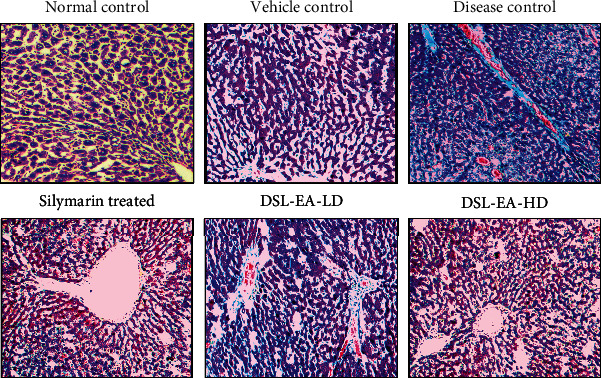
Masson's trichrome staining of the liver tissues. The extracts markedly improved the histological parameters and inhibited the liver fibrosis compared to the disease control.

The excessive accumulation of collagen and subsequent liver damage, i.e., fibrosis, was confirmed by Masson's trichrome staining. The results were in complete accordance with H&E-stained slides of liver tissue. Marked reduction in accumulation of extracellular matrix proteins (collagen) was observed in DSL-EA-HD-treated rats and likewise in the positive control group. Low dose of the extract also resulted in alleviating the injury caused by CCL_4_ compared to the disease control group as shown in Figure 5.

#### 2.4.6. Effect on Nrf2 and iNOS Expression Using Immunohistochemistry

**Figure 6:**
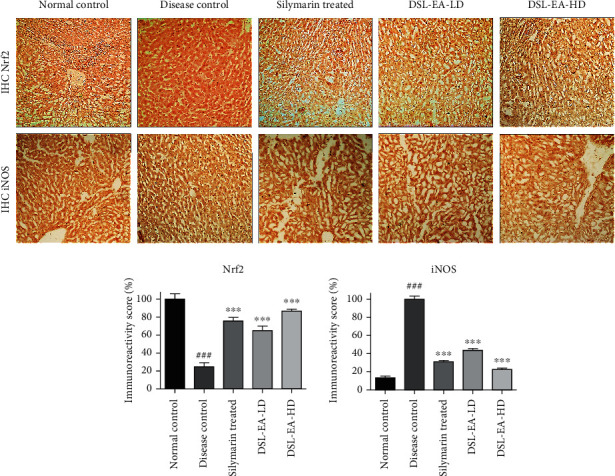
Effect of the DSL-EA-LD and HD treatment on expression level of nuclear factor erythroid 2(Nrf2) and inducible nitric oxide synthase (iNOS) in the liver tissue using immunohistochemistry analysis. The DSL-EA-LD and HD induced the expression of Nrf2 while inhibited the iNOS expression compared to the disease control. IHC: immunohistochemistry. All values are expressed as mean ± SEM (*n* = 6), ^∗^*P* < 0.05, ∗∗*P* < 0.01, and ^∗∗∗^*P* < 0.001 as compared to the disease control group.

The expression of nuclear factor erythroid 2(Nrf2) and inducible nitric oxide synthase (iNOS) was investigated using immunostaining. There was marked reduction in Nrf2 expression in CCL_4_-treated disease control group confirming the diminished resistance to oxidative stress. The expression level of iNOS on the other hand was increased due to obvious signs of tissue damage in the disease control rats. As shown in Figure 6, DSL-EA treatments have restored the normal Nrf2 and iNOS expression levels in a significant manner (*P* < 0.05, 0.01, and 0.001). As observed in hematological and biochemical investigations, the effect was dose dependent, with DSL-EA-HD- and silymarin-treated groups revealing identical immunoreactivity scores (Figure 6).

## 3. Discussion

Medicine derived from natural origin has gained immense interest and popularity in recent years owing to their great potential to prevent and treat several health issues linked to oxidative stress in a much safer and effective way [21]. Plants are considered the earliest source of drug discovery, and plant-based drugs have played a vital role in the healthcare system around the world [22]. Most medicinal plants have been known to be useful in mitigating more than one disease condition. This is because plants do possess a cocktail of constituents each possessing its own pharmacological effects. These constituents act via diverse mechanisms, and some of these have synergistic effects, while others have distinct therapeutic effects elicited through numerous receptors. Many such constituents are known to possess anti-inflammatory actions and are capable of repairing the inured cells or limiting the deleterious effects of inflammatory products [23].

*Datura stramonium* was chosen for the current study based on evidence regarding its reputation as a potential source of anti-inflammatory agents [20, 24]. In the initial phase of our study [16], pharmacologically significant secondary metabolites were estimated in the ethyl acetate leaf extract (DSL-EA) of selected plant followed by *in vitro* antioxidant and anticancer assessment. The toxicity profile of the extract was checked, and once the safety index was established, the extract was utilized in assessing the *in vivo* anti-inflammatory activity using CCl_4_-induced hepatic injury model in the rats.

Phytochemical investigations of DSL-EA revealed the presence of phenolics and flavonoids as reported in our previous paper [16], and these polyphenolic moieties play a vital role in the overall antioxidant defense mechanism of plants owing to the presence of hydroxyl, methoxy, ketonic, and phenolic functional groups in their chemical structure [25]. These compounds are widely reported to have free radical scavenging and lipid peroxidation inhibitory activities [16]. Polyphenolic compounds have a wide array of pharmacological benefits including but not limited to their anticancer, antioxidant, anti-inflammatory, antiangiogenic, and tumor suppressive actions [26–30]. Moreover, the hepatoprotective effect of polyphenols, i.e., gallic acid, rutin, apigenin, and catechin, has been widely documented in multimodal preclinical *in vitro* and *in vivo* studies [31–34].

Advancements in molecular biology have strengthened and rejuvenated the research interest of scientists in already established evidence of close association between inflammation and cancer [35, 36]. Once triggered by endogenous or exogenous stimuli, restoration of normal tissue physiology and elimination of toxins are achieved by inflammatory response of the body. However, in chronic cases, it can lead to serious disease conditions, the most worrying pathological state being cancer. Epidemiological data also indicates that one-fourth of all cancers are linked to chronic unresolved inflammatory disorders [37]. Chronic inflammatory disorders may pave way to situations that foster genomic lesions and cancer progression. The host combats such deleterious insults by numerous mechanisms, one prominent effector mechanism is the generation of free radicals, i.e., reactive oxygen species (ROS), superoxide (O₂-•), hydroxyl radical (OH•), and reactive nitrogen species (RNS), i.e., nitric oxide (NO•) and peroxynitrite (ONOO-). These molecules are produced by the activities of host enzymes, i.e., NADPH oxidase, myeloperoxidase, and nitric oxide synthases. These enzymes are regulated by signaling pathways involved in inflammation. Unregulated ROS and RNS production leads to oxidative stress and damage to DNA bases that ultimately results in elevated risk of DNA damage [38].

The nitric oxide scavenging potential of DSL-EA in LPS-challenged murine macrophages was estimated, and at concentration of 2.5-20 *μ*g/mL, significant inhibition of NO production was observed. Macrophages activated by immune response generate NO at a higher rate at inflammatory sites which then play a major role as immune regulators and neurotransmitters in various tissues [39, 40]. Scavenging the excessive NO radicals thus constitutes a prominent therapeutic approach for curbing inflammatory disorders.

The toxicity profile of DSL-EA was evaluated, and no significant cytotoxic action was observed against isolated normal human lymphocytes [16]. Cytotoxicity was further gauged by quantifying DSL-EA's action against macrophages isolated from rat peritoneum. As discussed in the Results, even at the highest used concentration, DSL-EA did not kill the isolated macrophages which further proves the selective nature of its cytotoxic action. Keeping in view the escalating demand to discover new anti-inflammatory and anticancer drug moieties, it is quite imperative to identify potent molecules with clinically proven safety profile.

Following *in vitro* screening of the extract, acute toxicity was assessed in the rats at doses ranging from 150 to 2000 mg/kg. Observation of no harmful and damaging effects on any of the groups over a period of two weeks further confirmed the safety of DSL-EA within the specified dose range, and it led to the designing of noninvasive single day and subchronic anti-inflammatory *in vivo* assays. Two of the most widely recognized mice models to evaluate the anti-inflammatory potential of potential medicinal agents are carrageenan-induced paw edema and croton oil-induced anal edema inhibition tests [41]. Mediators effecting acute inflammatory responses generally work in three different phases with histamine and serotonin been released in first phase (first 1.5 hr), and second phase involves bradykinin release (1.5-2 hr), while prostaglandins are involved in the third and last phase (2.5-6 hr) [42]. Carrageenan-induced edema is a biphasic model, and edema induction in the first two hours is due to bradykinin, serotonin, and histamine release, while in the latter stages (3-5 hr), prostaglandins are primarily responsible for edema [43]. Moreover, croton oil-induced inflammatory responses are primarily characterized by edema, greater vascular permeability, neutrophil infiltration, and prostaglandin production [44]. Considerable reduction in edema volume was observed at the 4^th^ hour following administration of predetermined doses of DSL-EA in both models of inflammation used in the current study. The anti-inflammatory response was in a dose-dependent manner, and previously published data support our findings [20, 42]. It can be inferred from the pattern of observed edema inhibitory action that the leaf extract has a tendency to limit the production of certain inflammatory mediators and proinflammatory cytokines.

Inflammation is reckoned to play a critical role in promoting susceptibility to depression, so treating inflammation, regardless of its type and cause, can be of great therapeutic benefit in improving the overall health status [45]. There is ample data to support the notion that depression is accompanied by elevated oxidative and nitrosative stress, and it also has a strong association with chronic inflammatory response [46, 47]. Several pathways are involved in carrying signals to the brain in the event of peripheral inflammation, i.e., cytokine transport system, vagus nerve and leaky regions in the blood-brain barrier [48], and peripheral cytokines are then disturb the synthesis and reuptake of neurotransmitters including, serotonin, dopamine, and norepinephrine [49]. In the current study, tail suspension test was used to predict the effect of DSL-EA on the behavior of test animals when they were exposed to testing conditions. Tail suspension test is a commonly used behavioral test in rodents and is used to assess the clinical effectiveness of antidepressant agents [50]. The efficacy of test samples is usually scaled by observing the reduction in immobility time, i.e., the state of helplessness displayed by test animals. DSL-EA HD, when compared with the positive control (fluoxetine), displayed mild effect, while slight reduction in the immobility time was observed in the low-dose group animals. *D. stramonium* has reported ethnomedicinal uses in epilepsy and depression when used internally, while in the form of ointments, it was utilized by ancient communities in rheumatism and burns [51], but there is lack of latest comprehensive data about its antidepressant potential. Hot plate test is a prominent nociceptive test using thermal stimuli to assess the centrally mediated analgesic action of test samples [52]. Scientific evidence supports the notion that drug molecules causing increase latency period possibly possess central analgesic activity [53]. Ethnopharmacological use of *D. stramonium* leaves as an antinociceptive agent was further validated by conducting the hot plate assay using mice model. Moderate elevation in latency period was observed in the case of DSL-EA HD, while low dose revealed slight antinociceptive response as per readings taken at 4^th^ hour of the experiment. *D. stramonium* leaves have been used traditionally for the management of pain externally as topical preparations [54]; the plant also has reported pharmacological use as an analgesic in variety of inflammatory disorders and pain [19, 55]. Our observations have further reinforced this ethnomedicinal and folklore use of *D. stramonium*. Promising results in toxicity studies and acute *in vivo* assays braced the plan to perform chronic *in vivo* anti-inflammatory assay using carbon tetrachloride (CCL_4_) as inducer of hepatic injury. The liver is continuously exposed to exogenous moieties derived from food, drugs, chemicals, and microbiota in the gut even under normal physiological conditions. Liver parenchymal and nonparenchymal cells are susceptible to harm instigated by oxidative stress, and prolonged stress can lead to changes in the composition of parenchymal cells as well as hepatic extracellular matrix. The cascade of events then causes recruitment of inflammatory and immune cells at the site of injury which further result in activation of nonparenchymal cells, i.e., stellate and hepatic Kupffer cells [56]. This is followed by a significant elevation in the levels of cytokines, chemokine, and growth hormones leading to liver fibrosis, the gateway to numerous hepatic abnormalities including HCC [57]. Regardless of the intrinsic dissimilarities between numerous etiological factors responsible for fibrosis, cirrhosis, and HCC, the preservation of wound healing response triggered by parenchymal cell death and the subsequent inflammatory cascade is a common denominator [58]. CCL_4_ is a toxicant linked to liver damage via generation of oxidative stress and injury of cellular components [59]. In the current study, the effect of DSL-EA extract in CCL_4_-induced liver inflammation was assessed through a series of hematological, biochemical, enzymatic, and histological studies. Moreover, immunohistochemistry studies were also incorporated to further scrutinize the anti-inflammatory potential of tested sample. Hematological investigations are a useful prognostic tool for underlying inflammatory conditions and consequent oxidative stress in vital organs including liver. CCL_4_ is known to cause hematological aberrations including lysis of RBCs and anemia following its metabolism and ROS production [60]. Distinctly decreased RBCs and hemoglobin levels and higher WBCs count in the disease control rats indicated the harm toxic effects of chemical toxin used in the study. The DSLA-EA-treated groups revealed almost identical results to the positive control “silymarin” used in the study. The presence of pharmacologically significant secondary metabolites including polyphenols, terpenoids, withanolides, steroidal glycosides, and alkaloids might be responsible for the vasotonic effects of DSL-EA. Estimation of serum levels of enzymes, i.e., ALT, AST, and ALP, are considered as important indicators of the functional integrity of hepatocellular membranes. These are cytosolic enzymes and seep out into the plasma in the event of hepatic injury accounting for their raised levels in the serum [14, 61] as observed in the disease control rats in our study due to CCL_4_ intoxication. Elevated bilirubin level in serum is also an indication of underlying liver damage and subsequent obstruction in bile excretion, thus serving as a useful confirmative test [62]. Similarly, reduced level of serum albumin is also an indication of ROS-mediated inflammatory condition within the body due to protein oxidation and lipid peroxidation-type reactions [63]. DSL-EA's effect on the said parameters was quite remarkable, and there was significant difference between the findings of the DSL-EA-treated groups and disease control rats. While the mechanism of restorative effect on liver functionality is not clear, this might be accredited to free radical scavenging and lipid peroxidation inhibitory potential of polyphenolic compounds present in the plant [26]. Oxidative stress due to CCL_4_ intoxication damages the antioxidant defense mechanism by deactivating the cellular antioxidant enzymes. Trichloromethyl peroxy radicals (CCl_3_OO‧) derived from CCl_4_ cause lipid peroxidation and inhibition of oxidative enzymes, thus leading to over accumulation of O_2_‧- and H_2_O_2_ resulting in massive outpouring of free radicals causing hepatic injury [64]. The major antioxidant enzymes responsible for neutralization of free radicals are GST, SOD, CAT, and POD. Moreover, a nonenzymatic antioxidant, GSH, also plays a major role in shielding hepatocytes by scavenging hydrogen peroxides and lipid peroxides as well as through its role as a substrate in catalytic action of glutathione peroxide [65]. The restoration of mentioned antioxidant defense system by DSL-EA, specifically in high-dose group, was remarkable, and the results were almost comparable to positive control used in the study. The findings of *in vitro* antioxidant and anti-inflammatory assays in our study are further validated with these results. Medicinally important phytoconstituents of *D. stramonium* leaf, i.e., terpenes [66], essential oils [42], polyphenols, steroids, tannins [67], and steroidal glycosides [68], might be responsible for the restorative effects observed in our study.

Oxidative stress markers addressed in the current study are NO, MDA, and MPO levels. A debilitated antioxidant defense setup results in dire consequences including greatly increased lipid peroxidation and loss of cellular membrane integrity. There are several end products of lipid peroxidation including MDA. Furthermore, there exists a close association between MPO enzyme level and oxidative stress [69]. Increase in MPO level serves as an indirect indicator of neutrophilic infiltration and inflammation [70]. Finally, in inflammatory conditions, NO is synthesized in excessive amount, surpassing the normal physiological NO level by almost 1000 folds, and this exceedingly higher NO production can result in ROS-mediated tissue damage [71]. Estimating the effect of DSL-EA on the levels of abovementioned markers thus provided a stout indication of its overall anti-inflammatory potential. The results observed were in accordance with the findings of biochemical and antioxidant enzyme level. MDA and MPO levels in liver tissue were greatly elevated, and NO production was also observed to be higher both in plasma and liver tissue of disease control rats, indicating the antioxidant defense system to be in dire straits due to CCl_4_ intoxication. Considerable reduction in % concentration of MDA and NO was observed in the DSL-EA-treated groups (more effective reduction in high-dose group). Our results further support the findings of previously reported potential of *D. stramonium* leaf extract in curbing oxidative stress [16]. MPO level was also reduced in the treatment groups as compared to significantly elevated level in the disease control rats. Biotransformation of CCl_4_ is carried out by endoplasmic reticulum (ER) which is one of the chief cellular organelle responsible for normal cellular functions [72, 73]. CCl_4_ is known to disrupt the normal function of ER within the hepatocytes leading to centrilobular necrosis and fatty degeneration of the liver [74]. CCl_4_ intoxication is normally associated with damaged ER and cellular membrane, immune cell infiltration, and necrosed hepatocytes, and all these collectively result in severely disfigured hepatocyte ultrastructure [75]. In the present study, H and E staining of the liver tissue of disease control group revealed immune cell infiltration, fibrosis, necrosed hepatocytes, and edema. DSL-EA in a manner identical to silymarin restored the normal histoarchitecture of liver tissues. The findings are in true agreement with a range of investigations performed in the current study proving significant anti-inflammatory action of the plant extract. Masson's trichrome staining is used effectively to measure the extent of liver fibrosis and necrosis by detecting collagen in liver tissues [76]. The presence of hyperplastic fibrous tissue due to CCl_4_-induced liver damage was confirmed by Masson's staining. Clear improvement was observed in the DSL-EA-treated groups in terms of detected collagen content and fibrosis, further confirming the results of preceding investigations done in the current study. Numerous *in vivo* studies have proved the pivotal role played by Nrf2 in inflammatory diseases including liver damage. Nrf2^−/−^ animals used in these studies have shown aggravated tissue damage and symptoms of inflammation. It is thus postulated that Nrf2 signaling pathway has a definite protective role in inflammatory disorders [77, 78]. Nrf2 signaling helps in curbing the inflammatory insults by regulation of endogenous antioxidant enzymes and proinflammatory cytokines [78]. The progression of an early phase liver injury to fibrosis usually is preceded by an inflammatory phase with building up of continuous oxidative stress, and under these circumstances, there is upregulation of iNOS and consequential generation of greater amounts of NO [79]. In our study, reduced Nrf2 and elevated iNOS expressions were observed in liver tissue of the CCl_4_-treated disease control rats using immunohistochemistry analysis. Nrf2 expression was elevated by DSL-EA in a dose-independent manner and both low- and high-dose groups exhibited identical improvement. The expression level of iNOS on the other hand was distinctly reduced by high dose of the extract and the positive control used in the study.

Outcomes of our study have scientifically validated the folkloric usage of *D. stramonium* in inflammatory diseases. Notwithstanding the fact that animal models have several common features to human physiology and much has been learned about human development by using animal models, due diligence exercise must be undertaken while extrapolating outcomes of an animal model to clinical studies.

## 4. Materials and Methods

### 4.1. Plant Collection and Preparation of Extract

*D. stramonium* was collected in August 2016 from district Mansehra in Khyber Pakhtunkhwa province of Pakistan. The plant was identified by Prof. Dr. Rizwana Aleem Qureshi, Department of Plant Sciences, Faculty of Biological Sciences, Quaid-i-Azam University, Islamabad, Pakistan. Following identification, a dried sample of *D. stramonium* was deposited at the Herbarium of Quaid-i-Azam University, Islamabad, with voucher number PHM-504. The plant was washed with water, segregated into parts (root, stem, fruit, and leaf), and shade dried at a properly ventilated place. Fully dried plant parts were grinded into fine powder and weighed. Ultrasonication-assisted maceration was performed for successive extraction using four different solvents with increasing order of polarity (n-hexane, ethyl acetate, methanol, and distilled water). The entire extraction process was repeated twice. Prior to rotary evaporation, the extracts were filtered using Whatman No. 1 filter paper and later dried in vacuum oven at 40°C. Numerous bench top assays were performed by weighing out and preparing samples of various concentrations. The extracts were stored at -20°C until further use.

### 4.2. Chemicals and Reagents

All chemicals and reagents consumed in our study were of analytical grade and were purchased from authentic vendors. Solvents, i.e., ethyl acetate and dimethylsulfoxide (DMSO), were purchased from Merck (Darmstadt, Germany). Potassium dihydrogen phosphate, dipotassium hydrogen phosphate, ferrous chloride, sodium hydroxide, aluminum chloride, ascorbic acid, quercetin, gallic acid, rutin, caffeic acid, kaempferol, myricetin, and (+)-catechin were acquired from Sigma-Aldrich (Steinheim, Germany). Folin-Ciocalteu reagent was purchased from Sigma-Aldrich (Steinheim, Germany). Sodium carbonate, sulphuric acid, hydrogen peroxide, potassium ferricyanide, sodium dihydrogen phosphate, and disodium hydrogen phosphate were purchased from Merck KGaA (Darmstadt, Germany). Tween 80, thiobarbituric acid, trichloroacetic acid, ferric chloride, and phenazine methosulphate were acquired from Sigma (Chemicals Co. St. Louis, USA). The chemicals and standard drugs were freshly made before use. Primary and secondary antibodies used for Nrf2 and iNOS proteins were procured from Santa Cruz (Santa Cruz Biotechnology, Inc.). All other chemicals were obtained from Sigma (Chemicals Co. St. Louis, USA).

### 4.3. Animals

Sprague-Dawley rats and BALB/c mice were acquired from the National Institute of Health, Islamabad, Pakistan (NIH).

### 4.4. *In Vitro* Anti-inflammatory Assay

#### 4.4.1. Nitric Oxide Scavenging Potential

The selected extract was also analyzed for its nitric oxide (NO) scavenging potential in murine macrophages using the Griess reagent method reported previously [80]. The experiment was performed in a 96-well plate, isolated macrophages were seeded at a density of 1 × 10^6^ per well, and the plate was kept at 37°C for 24 hr in a 5% CO_2_ incubator. The cells were then treated with various concentrations (2.5 to 20 *μ*g/mL) of test samples and controls and incubated for another two hours under similar conditions. It was followed by addition of 1 mg/mL LPS (10 *μ*L) to the medium, and the cells were incubated for further 18 hr. The Griess reagent (100 *μ*L) was then mixed with an equal volume of culture medium and absorbance was recorded at 540 nm. The positive control used in this assay was piroxicam, while LPS was taken as a blank. Quantification of nitrite in the media was done using the sodium nitrite standard curve (*y* = 0.0083*x* + 0.0017, *R*_2_ = 0.9999). The inhibitory capacity of DSL-EA against LPS-induced NO generation by macrophages was obtained from the estimated value of “*x*” in the regression equation.

Ethyl acetate leaf extracts of *D. stramonium* were selected for estimation of *in vivo* anti-inflammatory activity based on its pharmacologically significant primary and secondary metabolites as well proficient *in vitro* antioxidant and NO scavenging capacity.

### 4.5. Toxicity Assays

(2.4.1) Lymphocyte and Macrophage Toxicity Assays

Toxicity against human lymphocytes used in the study was checked as reported in our previous publication [16].

The toxicity was also assessed against macrophages isolated from rat peritoneum. The percent cell viability assessment of DSLA-EA was done using MTT assay [81]. The experiment was performed in a 96-well plate, and isolated macrophages were seeded at a density of 1 × 10^6^ per well. The cells were then exposed to different concentrations (2.5 to 20 *μ*g/mL) of test samples and controls and incubated at 37°C in a 5% CO_2_ incubator. Following 24 hr incubation period, 20 *μ*L MTT solution (1 mg/mL normal saline) was introduced into each well and kept in incubator under identical conditions for 2 hr. Succinate dehydrogenase in mitochondria of alive cells converted MTT into formazan crystals which are purple in color. These crystals were taken and solubilized in DMSO (100 *μ*L). The absorbance of resulting solution was estimated at 570 nm. Doxorubicin (1-100 *μ*M) was the positive control, while PBS was used as a negative control in this assay. Percent cell viability was determined using the following formula.

(1) $$\begin{array}{c} \%\text{cell viability}=\frac{\text{Ab sample}-\text{Ab blank}}{\text{Ab conrol}-\text{Ab blank}}\text{x }100. \end{array}$$

### 4.6. Acute *In Vivo* Studies

#### 4.6.1. *In Vivo* Acute Toxicity in Rats

To determine the safety profile of extract, acute toxicity assay was performed. Sprague-Dawley rats (6-8 weeks old), weighing approximately 150-250 g, were randomly divided into test and control groups (*n* = 6). Test groups received single booster oral dose of different strengths (150, 300, 500, 1000, and 2000 mg/kg). The control group received normal saline (10 mL/kg). Animals were kept under observation for two weeks, and any symptoms of toxicity and/or deaths were recorded. The guidelines provided by the Organization for Economic Cooperation and Development (OECD) were followed during the study.

#### 4.6.2. Noninvasive Acute *In Vivo* Tests

The acute *in vivo* activity indicated that the extract was safe to administer in the range of 150-2000 mg/kg concentrations. Therefore, DSL-EA in two different doses, i.e., 150 mg/kg (low) and 300 mg/kg (high), dissolved in 10% DMSO in olive oil were used to determine the acute *in vivo* anti-inflammatory, antidepressant, and antinociceptive activity in BALB/c mice. Healthy male mice, 6-8 weeks old, weighing between 25 and30 g were used in the study. Mice were kept in standard metallic cages and provided with standard diet and water *ad libitum*. Mice were randomly divided into five groups with six mice in each group, and their description is given as follows.

- Group I: normal control (untreated, standard food only)
- Group II: vehicle control (10% DMSO in olive oil)
- Group III: positive control (standard drug specific for each test)
- Group IV: DSL-EA LD (DSL-EA [150 mg/kg])
- Group V: DSL-EA HD (DSL-EA [300 mg/kg])

The experimental details of each noninvasive experiment are provided in the following section.

*(1) Carrageenan-Induced Hind Paw Edema Test*. The experiment was carried out by following a previously reported procedure with slight modifications [82]. The controls and sample groups were given oral doses of respective samples one hour before the administration of 0.05 mL of carrageenan (1% in sterile water for injection) into the subplanter region of right hind paw. Ibuprofen (the positive control) was also given orally at a dose of 10 mg/kg. Edema volume was estimated by measuring the thickness of right hind paw immediately after the carrageenan injection. Measurement of paw thickness was done at regular intervals for up to 4 hours. The results were expressed as percent edema inhibition using the following formula.

(2) $$\begin{array}{c} \text{EV}=\text{PVa}-\text{PVi}, \end{array}$$

where EV is edema volume, PVi is initial paw volume (before carrageenan administration), and PVa is paw volume following carrageenan injection.

(3) $$\begin{array}{c} \text{Percent edema inhibition}=\left[\frac{\text{EVc}-\text{EVt}}{\text{EVc}}\right]\text{ x }100, \end{array}$$

where EVc is edema volume of control mice and EVt is edema volume of test sample mice.

*(2) Croton Oil-Induced Anal Edema Inhibition*. Anti-inflammatory potential of DSL-EA was further evaluated by estimating the croton oil-induced anal edema inhibitory response in Balb/c mice. A previously reported procedure was followed [83], and as elaborated previously, different control and treatment groups received respective doses of standard drug and test samples one hour before the induction of acute inflammation. A cotton swab swabbed in the inducer was introduced gently into the anus of the mice for 10 seconds. The inducer used in this assay was croton oil (200 *μ*L of 6% croton oil in diethyl ether). The anal edema was measured using a Vernier caliper each hour for four hours following induction. The anti-inflammatory potential of samples, if present, was indicated by reduction in anal edema of treatment groups when compared with the control group. The formula used to calculate percent inhibition in anal edema is given as follows.

(4) $$\begin{array}{c} \%\text{inhibition}=\left(\frac{\text{Control animal edema volume}-\text{test group edema volume}}{\text{Control animal edema volume}}\right)\text{x }100. \end{array}$$

*(3) Tail Suspension Test*. Tail suspension test has been effectively used to determine the antidepressant action of tested samples. Slightly modified version of a previously reported protocol was followed in the current study [50]. One hour after administration of extracts and specified samples to relevant groups, mice were individually suspended from their tails using an adhesive tape placed 1 cm from the tip of the tail. The mice were suspended in such a way that they were at 7.5 cm above the surface of a working table. The experiment was carried out in a quiet room and the total duration of the test was 6 minutes. Immobility time was recorded for each group, i.e., that situation during the study when the mice stayed passively hung without any motion or urge to move. The positive control used in this test was fluoxetine given intraperitoneally (i.p) (20 mg/kg). Samples possessing antidepressant action are expected to reduce the immobility time of the mice.

*(4) Hotplate Analgesic Assay*. Hotplate analgesic assay is a well-established and reliable procedure to determine the antinociceptive response in rodents [84]. Before administering the test samples, the mice were individually placed on a heated pate set at 55 ± 2°C and the paw licking and jumping actions were recorded, the time at which the mice start showing these responses was recorded (Ta), and an average of two readings is normally taken. A cutoff time was set at 30 seconds to prevent tissue damage. One hour after administration of test samples and controls to relevant study groups, each animal was placed again on the heated plate and reaction time, i.e., the time taken to initiate jumping and paw licking while being on the hotplate, was noted (Tb). The reaction times were recorded at regular interval of one hour for up to 4 hours. The positive control used in this test was tramadol (12.5 mg/kg via i.p route). The results are expressed as mean ± SD of percent analgesia. The percent analgesic or antinociceptive activity was estimated using the following formula.

(5) $$\begin{array}{c} \text{Percent analgesic activity}=\frac{\text{Tb}-\text{Ta}}{\text{Ta}}\text{ x }100. \end{array}$$

### 4.7. *In Vivo* Study (CCL_4_-Induced Toxicity in Sprague-Dawley Rats)

#### 4.7.1. Experimental Design

*(1) Animal Model*. Thirty-six (36) male Sprague-Dawley rats, 6-8 weeks old with weights in the range of ~150-250 g, were selected for the current study. Aluminum cages with wood shavings as bedding were used to retain the animals, and the temperature was maintained at 25 ± 1°C and air humidity at 45 ± 5% with a 12 h light/dark cycle. The rats were fed with standard feed and water ad libitum prior to their experimental use.

*(2) Experimental Protocol*. The rats were randomly divided into six groups after a one-week acclimatization period, each group consisted of six rats. Predetermined high and low doses (as indicated by acute toxicity study) of DSL-EA and standard drug used as positive control were orally administered via sterile oral gavage to specified groups. The maximum volume to be administered orally was kept constant at 1 mL. The vehicle used for making suspensions of the extract was 10% DMSO in olive oil. The study had four controls, i.e., normal, vehicle, disease and positive control groups. All doses to respective groups were given in the morning on alternate days.

- Group I: normal control (untreated, standard food only)
- Group II: vehicle control (10% DMSO in olive oil)
- Group III: disease control (1 mL/kg of 30% CCL_4_ in olive oil)
- Group IV: positive control (1 mL/kg of 30% CCL_4_+silymarin [50 mg/kg] dissolved in the vehicle)
- Group V: DSL-EA LD (1 mL/kg of 30% CCL_4_+DSL-EA [150 mg/kg])
- Group VI: DSL-EA HD (1 mL/kg of 30% CCL_4_+DSL-EA [300 mg/kg])

**Figure 7:**
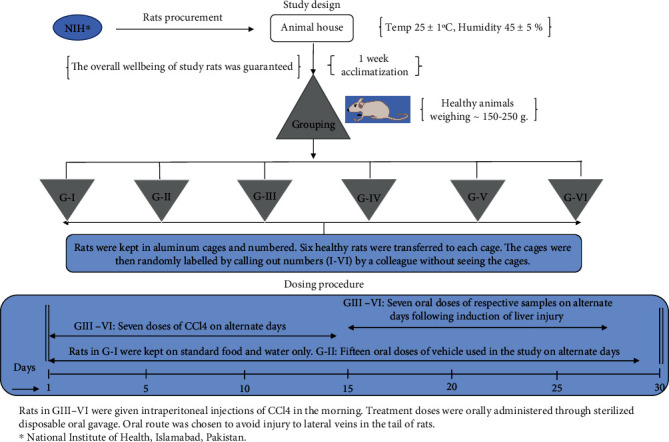
A schematic diagram of study design and experimental timeline of the *in vivo* anti-inflammatory assay performed using male Sprague-Dawley rats. Clip art images in the figure were made using ChemDraw Professional v20.0.

The current *in vivo* experiment lasted for 30 days. Group I rats received no treatment of any kind and were fed and watered ad libitum. Group II animals were given oral doses of the vehicle used in the study to assess any effect it might have on experimental rats. The rats in groups III-VI received intraperitoneal injections (7 doses) of the inducer (30% CCL_4_ in olive oil) used in current study. Following disease induction, the rats in groups IV, V, and VI were given 7 doses of silymarin, DSL-EA LD, and DSL-EA HD, respectively, on alternate days. The experiment ended on 29^th^ day and thereafter the rats were kept for 24 hrs before sacrificing and taking blood, serum, and tissue samples for analysis. The study design is elaborated further in Figure 7.

#### 4.7.2. CCL_4_-Induced Toxicity

As discussed previously, the rats used in the study were randomly distributed into 6 groups, and CCL_4_ model was adopted to induce liver injury [14, 85]. Hepatotoxicity was induced by injecting 0.2 mL CCL_4_ into the peritoneal cavity of the rats in groups III-VI on alternate days.

#### 4.7.3. Collection of Blood Samples and Serum Separation

At the end of the assay, the rats were unfed for 24 h, anesthetized by chloroform inhalation, and subsequently euthanized by cervical dislocation. The blood was collected from abdominal aorta for the assessment of hematological and biochemical parameters. Serum was separated from the collected blood samples by centrifugation for 15 min at 4°C (6000 rpm). Serum was stored at −20°C until analyzed for biochemical tests.

The excised and flash frozen liver portions of the rats from each study group were homogenized using 10× buffer (100 mM potassium phosphate buffer and 1 mM ethylenediaminetetraacetic acid; pH 7.4). Homogenates were then centrifuged for 30 min at 4°C (12,000 × *g*). The resultant supernatant was carefully separated and used for the analysis of endogenous antioxidant enzymes and numerous biochemical parameters.

#### 4.7.4. Estimation of Hematological Parameters

The levels of red blood cells (RBC) and white blood cells (WBC) were estimated by using Neubauer hemocytometer (Feinoptik, Germany). Hemoglobin level was measured with the aid of Sahli's hemoglobin meter. Differential WBC count (% neutrophils, monocytes, eosinophils, and basophils) was also determined following a previously reported method [86].

#### 4.7.5. Assessment of Biochemical Parameters

For the analysis of biochemical parameters, serum separated from the blood samples was used for analysis of alanine transaminase (ALT), aspartate aminotransferase (AST), alkaline phosphatase (ALP), bilirubin, creatinine, and albumin using AMP diagnostic kits (Stattogger Strasse 31b 8045 Graz, Austria) as per the instruction of the manufacturers.

#### 4.7.6. Determination of Endogenous Antioxidant Enzymes and GSH

The level of endogenous antioxidant enzymes, i.e., glutathione S-transferase (GST), superoxide dismutase (SOD), peroxidase (POD), and catalase (CAT) as well as glutathione (GSH), in serum was found using a slightly modified version of a protocol reported earlier [14].

#### 4.7.7. Expression of Biomarkers of Oxidative Stress (MDA, MPO, and NO)

Concentration of one of the lipid peroxidation end products, i.e., MDA, in liver tissue was estimated using a slightly modified version of previously reported procedure [87]. Briefly, 0.25 mL tissue homogenate in PBS was incubated at 37°C for 1 h in a water bath. Incubation was followed by addition of 0.25 mL of 5% trichloroacetic acid (TCA) and 0.5 mL of 0.67% thiobarbituric acid (TBA) to the homogenates. Absorbance was recorded at 535 nm.

The MPO activity was estimated using hexadecyltrimethylammonium bromide (HTAB) buffer and o-dianisidine method described in a previously published report [88]. HTAB in 50 mM PBS having pH 6 was used to release MPO from the cells. The tissue was then subjected to freeze thaw cycle for three times and later centrifuged. The supernatant was then mixed with a mixture of hydrogen peroxide and o-dianisidine, and MPO activity level was recorded at 540 nm using a microplate reader.

The level of nitric oxide was assessed both in plasma and liver tissue homogenates. The Griess reagent method was followed and the absorbance was recorded at 540 nm using a microplate reader [89].

#### 4.7.8. Histological Investigation (Hematoxylin-Eosin and Masson's Trichrome Staining)

The evaluation of any changes in liver histology was carried out by employing paraffin-embedded staining protocols. Following dissection, liver tissue was fixed by using 10% buffered formaldehyde solution (pH 7.4) at room temperature for 12 h. The removal of traces of infiltrated wax and water was assured via numerous ethanol (50, 70, 90, and 100%) washings of the fixed tissue. Small pieces (3-5 *μ*m thickness) of the embedded tissue were sectioned to prepare slides and later stained with eosin and hematoxylin (H and E) [16]. The trichrome staining was also performed to assess fibrosis in liver tissue [87]. Lastly, the slides were examined under microscope (Nikon, Eclipse 80i Japan).

#### 4.7.9. Immunohistochemistry

The immunohistochemistry staining was performed to examine the effect of DSL-EA extract on nuclear factor erythroid 2 (Nrf2) and inducible nitric oxide synthase (iNOS) following liver injury caused by CCL_4_ toxicity. Paraffin-embedded staining protocol was followed. The tissue was washed with xylene and ethanol followed by treatment with proteinase-k, normal goat serum (NGS), and primary and secondary antibodies (Nrf2 and iNOS) [90].

### 4.8. Statistical Analysis

The data presented in the study was procured from experiments run in triplicate, and the results are expressed as mean ± SD. A one-way analysis of variance (ANOVA) was performed followed by Dunnett's test to determine the variability between test groups. Statistical significance was set at *P* < 0.05. The SPSS software (SPPS version 10.0, Chicago, IL) was used to analyze data.

## 5. Conclusion

Estimation of pharmacologically significant phytoconstituents and proficient results in the *in vitro* biological screening steered the comprehensive evaluation of anti-inflammatory potential of selected extract of *D. stramonium*. Noteworthy acute *in vivo* anti-inflammatory potential further supported the execution of subsequent chronic *in vivo* model. Findings of our study recommend that DSL-EA within a prudently calculated dosing window has the aptitude to restore liver functionality in the event of CCl_4_-induced damage. This claim is supported by results procured from a series of investigations including hematological, biochemical, liver enzymes level, oxidative stress markers, histological, and immunohistochemistry studies. The selected leaf extract of *D. stramonium* has effectively managed to curb the liver damage caused by CCL_4_ intoxication. Robust anti-inflammatory action of *D. stramonium* has validated its traditional use in numerous inflammatory conditions. More advanced studies for further elaboration of the molecular mechanism of its action will be the most logical extension of our preliminary research work.

## Figures and Tables

**Table 1:** Effect of the DSL-EA-LD and DSL-EA-HD on the complete blood profile.

Hematological parameters	Normal control (mean ± SEM)	Vehicle control (mean ± SEM)	Disease control (mean ± SEM)	Silymarin treated (mean ± SEM)	DSL-EA-LD (mean ± SEM)	DSL-EA-HD (mean ± SEM)
HGB (g/dL)	12.86 ± 0.45	12.67 ± 0.92	8.02 ± 1.02^###^	11.98 ± 0.087^∗^	10.34 ± 0.23^∗^	12.10 ± 1.03^∗^
RBC (×10^6^/mm^3^)	10.01 ± 0.34	10.2 ± 0.04	6.07 ± 0.32^###^	9.98 ± 0.22^∗^	8.78 ± 0.43^∗^	10.06 ± 0.23^∗^
WBC (×10^3^/mm^3^)	8.078 ± 1.21	8.11 ± 0.04	13.12 ± 0.03^###^	8.98 ± 0.03^∗^	9.43 ± 0.23^∗^	8.34 ± 0.32^∗^
Neutrophils (%)	33.7 ± 4.06	31.3 ± 1.07	62.04 ± 1.31^###^	45.8 ± 0.72^∗∗^	59.87 ± 1.23^∗^	36.14 ± 0.32^∗∗^
Monocytes (%)	5.83 ± 0.70	5.43 ± 0.70	12.12 ± 1.03^###^	7.09 ± 0.35^∗^	8.92 ± 1.12^∗^	7.64 ± 1.09^∗^
Eosinophils (%)	0.78 ± 0.10	0.73 ± 0.05	0.90 ± 0.22^###^	0.78 ± 0.12^∗∗^	0.852 ± 0.01^∗∗^	0.761 ± 0.05^∗∗^
Basophils (%)	0.45 ± 0.02	0.461 ± 0.12	0.81 ± 0.05^###^	0.57 ± 0.14^∗^	0.598 ± 0.051^∗^	0.521 ± 0.12^∗^

All values are expressed as mean ± SEM (*n* =6), ^∗^*P* < 0.05, ^∗∗^*P* < 0.01, and ^∗∗∗^*P* < 0.001 as compared to the disease control group. The results were analyzed by the two-way ANOVA followed by Dunnett's test. HGB: hemoglobin; RBC: red blood cells; WBC: white blood cells.

**Table 2:** Effect of the DSL-EA-LD and DSL-EA-HD on the biochemical parameters.

Biochemical parameters	Normal control (mean ± SEM)	Vehicle control (mean ± SEM)	Disease control (mean ± SEM)	Silymarin treated (mean ± SEM)	DSL-EA-LD (mean ± SEM)	DSL-EA-HD (mean ± SEM)
ALT (U/L)	25.32 ± 1.1	25.9 ± 1.50	188.73 ± 3.24^###^	56.45 ± 1.24^∗∗^	78.62 ± 2.81^∗∗^	51.32 ± 2.18^∗∗^
AST (U/L)	28.9 ± 1.53	30.1 ± 2.40	231.98 ± 2.32^###^	76.2 ± 4.12^∗∗^	96.71 ± 3.12^∗∗∗^	80.35 ± 4.00^∗∗^
ALP (U/L)	45.12 ± 2.10	47.56 ± 1.90	172.74 ± 4.41^###^	67.54 ± 1.21^∗∗^	92.42 ± 2.32^∗∗^	62.81 ± 2.90^∗∗^
Bilirubin (mg/dL)	1.023 ± 0.02	1.1 ± 0.011	2.99 ± 0.04^###^	1.870 ± 0.09^∗∗∗^	2.09 ± 0.087^∗∗∗^	1.54 ± 0.71^∗∗∗^
Albumin (g/L)	16.34 ± 1.32	15.32 ± 0.87	4.28 ± 0.09^###^	12.45 ± 0.10^∗∗^	8.12 ± 0.84^∗∗^	13.5 ± 0.09^∗∗^
Creatinine (mg/dL)	0.276 ± 0.03	0.284 ± 0.10	1.08 ± 0.04^###^	0.41 ± 0.089^∗^	0.73 ± 0.13^∗^	0.378 ± 0.10^∗^

Values are expressed as mean ± SEM (*n* = 6), ^∗^*P* < 0.05, ^∗∗^*P* < 0.01, and ^∗∗∗^*P* < 0.001 as compared to the disease control group. The results were analyzed by the two-way ANOVA followed by Dunnett's test. ALT: alanine transaminase; AST: aspartate aminotransferase; ALP: alkaline phosphatase; U/L: units per liter; mg/dL: milligrams per deciliter; g/L: grams per liter.

## Data Availability

The datasets presented in the current study are available from the corresponding author on reasonable request
